# Urethral instillation of chlorhexidine gel is an effective method of sterilisation

**DOI:** 10.1080/2090598X.2021.1956832

**Published:** 2021-09-16

**Authors:** Osama Shaeer, Amr Abdel- Raheem, Haitham Elfeky, Ahmad Seif, Tarek M. Abdel-Raheem, Amgad Elsegeiny, May Sherif Soliman, Emad B. Basalious, Kamal Shaeer

**Affiliations:** a Andrology Department, Kasr El Aini Faculty of Medicine, Cairo University, Cairo, Egypt; b Medical Physiology Department, Kasr El Aini Faculty of Medicine, Cairo University, Cairo, Egypt; c Clinical and Chemical Pathology Department, Kasr El Aini Faculty of Medicine, Cairo University, Cairo, Egypt; d Pharmaceutics and Industrial Pharmacy Department, Cairo University Cairo, Cairo, Egypt

**Keywords:** Penile prosthesis, chlorhexidine gel, infection, sterilisation, urethral injury

## Abstract

**Objective**: To examine the effectiveness of preoperative urethral sterilisation with chlorhexidine gel in rendering the urethra as sterile as the skin of the genital area, with the skin sterilised as per the International Society for Sexual Medicine guidelines for penile prosthesis implantation.

**Patients and methods**: A total of 111 male patients undergoing sterile andrological surgical procedures were divided into a control group (*N* = 61) and a chlorhexidine gel group (*N* = 50). Patients in the chlorhexidine group received urethral instillation with 6 mL of chlorhexidine preoperatively and on table. Patients from both groups received on-table skin preparation using povidone iodine and chlorhexidine povidone iodine. At the end of surgery, swabs were obtained from urethra and the penile skin. Skin and urethral swabs were compared for bacterial colonisation by culture and sensitivity.

**Results**: Of the 111 patients, 16 had urethral colonisation and 10 had skin contamination, and they were all in the control group. The most common organism detected in both the urethral and skin samples was coagulase-negative *Staphylococcus aureus*. Urethral colonisation was significantly greater in the control group compared to the chlorhexidine group, at 16/61 vs 0/50 (*P* = 0.001). Similarly, skin colonisation was significantly greater in the control group compared to the chlorhexidine group, at 10/61 vs 0/50, (*P* = 0.002).

**Conclusion:** Chlorhexidine gel is a powerful sterilising agent that will render the urethra sterile.

## Introduction

The introduction of penile implants has revolutionised the management of male erectile dysfunction (ED). However, a number of intraoperative complications may occur (such as bleeding, infection, and urethral injury) which, in turn, have a major impact on clinical outcomes and patient satisfaction. Patients undergoing penile implant surgery with fibrotic corpora have a higher risk of urethral injury [1,2].

The classic and conservative line of management for urethral injury is to abort the penile prosthesis (PP) implantation, leave a urethral catheter and return to the operating room for a secondary attempt after urethral healing [3,4].

However, this approach has the disadvantage that a delayed implantation procedure can be challenging due to penile fibrosis, which has a major impact on clinical outcomes and patient satisfaction [1]. This has led some authors to recently suggest primary repair of distal, mid and corporotomy related urethral injuries with simultaneous implantation. Such injuries are often identified during dilatation or irrigation of the corpora and can be accessed, depending on the location, through the same incision or by making a counter subcoronal incision with penile degloving or by performing a meatotomy [5,6].

Primary repair of urethral injury, with catheterisation or a suprapubic cystostomy, and implantation in the same session could help avoid the difficulty and complications resulting from delayed re-insertion (including re-injury of the urethra due to difficult dilatation, bleeding, false passage, sepsis, and urethral stricture). However, implant infection is more probable with immediate repair and same-session implantation as the penile urethra is not necessarily sterile [7,8].

Although data suggests that the majority of implant infections occur due to contamination from the skin flora, many implanters recommend performing a urine culture before the surgery because of the risk of ascending infection from the urethral flora [9].

This aim of the present study was to examine the effectiveness of preoperative urethral sterilisation with chlorhexidine gel in rendering the urethra as sterile as the skin of the genital area when sterilised as per the International Society for Sexual Medicine (ISSM) guidelines for PP implantation.

## Patients and methods

Ethical approval for the study protocol was granted by the Regional Ethics Committee of the Faculty of Medicine, Cairo University hospitals. A prospective study involving 111 male patients undergoing aseptic surgery (regardless of the procedure) was undertaken. The patients were divided into two groups: a control group (*N* = 61) and a chlorhexidine gel group (*N* = 50).

The inclusion criteria were male patients aged >25 years undergoing a sterile surgical procedure (surgeries not involving treatment of a septic focus or a source of infection); not necessarily PP implantation. The exclusion criteria included: patients not consenting to participation in the study, patients with contraindication for anaesthesia, or unwilling to undergo surgery, patients with history of urethral surgery, urinary calculi, urethral structure, or ongoing pyospermia, prostatitis or pyuria; patients undergoing non-sterile surgeries such as abscess drainage.

Preoperatively, urethral instillation with 6 mL of chlorhexidine gel was performed in the chlorhexidine group using a 10-mL syringe inserted for a short distance into the meatus (the evening before surgery, and upon surgery, and on-table). For the control group, instillation was not performed.

The chlorhexidine gel was prepared in the pharmaceutics and industrial pharmacy department with the following composition: 117.6 mg lidocaine hydrochloride, 3.1 mg chlorhexidine digluconate, 3.8 mg methyl hydroxybenzoate (E218), and 1.6 mg propyl hydroxybenzoate.

All surgeries were performed by a single team of high-volume implanters who followed the same preoperative hand scrubbing technique and intraoperative aseptic techniques as per good surgical practice. On the surgical table, for both groups, skin sterilisation was performed according to the ISSM guidelines for skin preparation before PP surgery (regardless of the type of surgery being performed). Shaving was done on table; Povidone iodine and chlorhexidine povidone iodine shampooing was used for all patients for 10 min. The preparation solution was then applied using friction, from the cleanest area and proceeded in a concentric fashion to the least clean area (the area with a lower bacterial count prepared first, followed by the area of higher contamination). The applicator discarded once the periphery has been reached. The prepared area of skin extended to an area large enough to accommodate potential shifting of the drape fenestration, extension of the incision, and potential for additional incisions. The solution was allowed to dry completely naturally [10].

After the conclusion of surgery, and with the patient on the operating table, the following swabs were obtained: a penile skin swab and a urethral swab. Swabs were obtained in the operating theatre under complete aseptic precautions and transported promptly to the laboratory for microbiological procedures.

Patients who have had a penile implant procedure were followed-up for 6 months in order to record the rate of penile implant infection.

### Laboratory methods

Swab samples were directly transported to the laboratory for immediate culture. Culture on blood agar base supplemented with blood, MacConkey agar, and chocolate agar was done followed by incubation at 37°C for 48 h. Plates were examined after 24 and 48 h, and microbial growth was identified using standard procedures. Lactose fermenter colonies were subjected to conventional biochemical reaction in the form of triple sugar iron, motility indole ornithine, lysine iron agar, urease, and citrate. Non-lactose fermenter colonies were subjected to oxidase; if negative, a Gram stain was done to exclude *Acinetobacter* (coccobacilli) and if Gram-negative bacilli were found, the previous biochemical reactions were performed. *Staphylococcus* colonies were identified further using the mannitol salt and the DNase agar. Quality control was done for performance of the different media and identification methods in accordance with applicable regulations and accreditation requirements with Clinical and Laboratory Standards Institute (CLSI) guidance, using American Type Culture Collection (ATCC) strains as control strains for susceptibility testing at appropriate intervals [11].

Skin and urethral swabs were compared for bacterial colonisation by culture and sensitivity testing across the two groups.

### Statistical methods

Results are expressed as mean (± standard deviation [SD]) or number. Comparison between variables in the two groups was performed using the unpaired *t*-test. Comparison between categorical data (number) in the two groups was performed using the chi-square test. The Statistical Package for the Social Sciences (SPSS®) version 20 (IBM Corp., Armonk, NY, USA) was used for data analysis. A *P* ≤ 0.05 was considered as statistically significant. Comparative analyses were performed twice: for the whole sample and for the sample after excluding the cases that showed skin contamination despite sterilisation.

## Results

A total of 111 patients were included in the study, with a mean (SD, range) age of 40.77 (13.66, 20–73) years. All procedures were conducted in a single high-volume andrology unit over a 4-month period from March 2019 to July 2019.

All patients had sterile urine, semen and expressed prostatic secretion cultures. There were no statistically significant differences between both groups in terms of age, diabetes, hypertension or smoking status. In all, 20 patients were diabetic (12 in the control group and eight in the chlorhexidine group). Eight patients were hypertensive: four in each group.

The most common surgeries performed for the control and chlorhexidine groups were PP implantation and varicocelectomy (Table 1).

**Table 1.** Surgical procedures in the study group

Procedure, *n* (%)	Control group (*N* = 61)	Chlorhexidine group (*N* = 50)
Varicocelectomy	17 (27.8)	18 (36.0)
Penile implant	25 (40.9)	13 (26.0)
Penile curvature	8 (13.12)	3 (6.0)
Testicular sperm extraction	5 (8.2)	10 (20.0)
Fracture Penis	2 (3.3)	0 (0.0)
Haematocelectomy	1 (1.6)	0 (0.0)
Hydrocelectomy	1 (1.6)	3 (6.0)
Epididymovasostomy	1 (1.6)	1 (2.0)
Orchidopexy for testicular torsion	1 (1.6)	2 (4.0)

A total of 16 patients showed urethral colonisation, 10 patients had skin contamination and seven had combined urethral and skin contamination. The most common organism detected in both the urethral and skin samples was coagulase-negative *Staphylococcus aureus* (Table 2).

**Table 2.** Contamination site and organisms identified in the control group (*N* = 61)

Contamination site and organism	*N* (%)
**Urethral colonisation**	**16 (14.4)**
Organism:	
Coagulase-negative *S. aureus*	14 (12.6)
Haemolytic *Streptococci*	1 (0.9)
Non-haemolytic *Streptococci*	1 (0.9)
**Skin colonisation**	**10 (9)**
Organism:	
Coagulase-negative *S. aureus*	8 (7.2)
Anthracoid	1 (0.9)
Non-haemolytic *Streptococci*	1 (0.9)

There was no statistically significant difference between the chlorhexidine and control groups for mean age or the prevalence of diabetes. Urethral colonisation was significantly greater in the control group compared to the chlorhexidine group, at 16/61 vs 0/50 (*P* = 0.001). Similarly, skin colonisation was significantly greater in the control group compared to the chlorhexidine group, at 10/61 vs 0/50 (*P* = 0.002). The control group still had a statistically significant higher rate of urethral colonisation even after excluding cases with combined skin and urethral contamination (seven cases).

The 111 surgeries performed included 38 primary penile implant insertion procedures (13 chlorhexidine group and 25 control group). There were no implant infections in the chlorhexidine group vs two PP infections in the control group, with one of the two patients being diabetic.

## Discussion

Moderate to severe ED affects 5–20% of men worldwide. It is estimated that by the year 2025, 322 million men will have ED [12,13]. ED is treated in a stepwise manner with penile implants offered to patients who fail to respond to conservative lines of therapy. Penile implants have high patient and partner satisfaction rates exceeding 80%; however, implant surgery is not free from complications such as infection and urethral injury [14,15].

Urethral injury occurs in 1–3% of cases [16,17]. Corporal fibrosis is the main risk factor for urethral injury. Corporal scarring causes difficultly in dilatation and carries the risk of corporal crossover and urethral injury [1,2]. Causes of corporal fibrosis include diabetes, intracavernosal injection therapy, priapism, and delayed re-insertion following removal of an infected penile implant [18,19]. The other cause of urethral injury is the modelling procedure performed after placing the implant in men with Peyronie’s disease in order to correct residual curvature [20]. The classic management of urethral injury during implant surgery involves aborting the procedure and delayed re-insertion after healing of the urethral injury as confirmed by urethrogram [3,4]. However, delayed re-insertion is not free from complications including re-injury of the urethra and even more difficult dilatation [7,8].

In the present study, we have demonstrated the possibility of male urethral sterilisation to be as sterile as the skin prior to PP implantation surgery by using chlorhexidine gel, which is available commercially as Instillagel® (Farco-Pharma GmbH, Gereonsmühlengasse, Köln, Germany). Instillagel has been used successfully for urethral catheterisation for many years [21].

The most common skin preparations used today include products containing iodophors or chlorhexidine gluconate (CHG). Chlorhexidine digluconate is excellent for Gram-positive bacteria, good for Gram-negative bacteria and viruses, and fair for fungi with an average duration of coverage of ~6 h [22].

We have prepared chlorhexidine digluconate gel in our Pharmaceutical Department according to the ingredients of Instillagel. We used it for urethral instillation before the surgery for 50 male patients undergoing aseptic surgery. The urethra and the skin were found to be completely devoid of any microbes (contrary to the controls). This proves the efficacy of chlorhexidine digluconate gel as a sterilising agent for the urethra. While in the control group (which comprised 61 patients who did not receive chlorhexidine gel per urethra), 16 cases had urethral contamination with different types of bacteria. Furthermore, the skin of 10 cases was also contaminated with different types of bacteria and seven cases had combined urethral and skin infection. This may suggest skin contamination from the urethra, as the main organism found in the urethra and on the skin was *S. aureus*.

Overall, PP infection occurs in <5% of cases [23]. The source of infection is usually from skin flora introduced at the time of the operation. The most common organism is *S. epidermidis* in both primary and revision surgery (although Gram-negative faecal pathogens can also cause infection). Other organisms implicated include *Pseudomonas aeruginosa, Escherichia coli, Serratia marcescens, Enterococcus* species, *Proteus mirabilis*, and methicillin-resistant *S. aureus* (MRSA) [24].

Therefore, urethral sterilisation with chlorhexidine gel decreases the risk of infection from the urethral catheter, which may displace the urethral pathogens into the operative field during PP surgery. This would decrease the overall infection rate of penile implantation surgery. Indeed, in the present series, 38 primary penile implant procedures were performed and over a 6-months follow-up period, none of the patients in the chlorhexidine group had a postoperative PP infection as opposed to two infections in the control group.

Furthermore, in the event of a urethral injury during PP surgery, the fact that the urethra is rendered sterile would enable the surgeon to perform primary repair of the urethral injury with simultaneous implantation.

However, the limitations of the present study are that the sample size was that of convenience conducted on the patients who were undergoing surgery at our centre throughout the study span. Furthermore, no urethral injuries were encountered in either group. As such, future more highly-powered multicentre studies are warranted.

## Conclusion

Intra-urethral instillation of chlorhexidine gel makes the urethra as sterile as the skin that was prepared as per the ISSM guidelines for skin preparation for PP surgery.
